# Calibration of a Multiphase Model Based on a Comprehensive Data Set for a Normal Strength Concrete

**DOI:** 10.3390/ma12050791

**Published:** 2019-03-07

**Authors:** Peter Gamnitzer, Martin Drexel, Andreas Brugger, Günter Hofstetter

**Affiliations:** Unit of Strength of Materials and Structural Analysis, Institute of Basic Sciences in Engineering Sciences, Innsbruck University, Technikerstr. 13, A-6020 Innsbruck, Austria; Martin.Drexel@uibk.ac.at (M.D.); Andreas.Brugger@uibk.ac.at (A.B.); Guenter.Hofstetter@uibk.ac.at (G.H.)

**Keywords:** model calibration, hygro-thermo-chemo-mechanical modelling, hardening concrete, desorption

## Abstract

Hygro-thermo-chemo-mechanical modelling of time-dependent concrete behavior requires the accurate determination of a large set of parameters. In this paper, the parameters of a multiphase model are calibrated based on a comprehensive set of experiments for a particular concrete of grade C30/37. The experiments include a calorimetry test, tests for age-dependent mechanical properties, tests for determining the water desorption isotherm, shrinkage tests, and compressive creep tests. The latter two were performed on sealed and unsealed specimens with accompanying mass water content measurements. The multiphase model is based on an effective stress formulation. It features a porosity-dependent desorption isotherm, taking into account the time-dependency of the desorption properties. The multiphase model is shown to yield excellent results for the evolutions of the mechanical parameters. The evolution of the autogenous shrinkage strain and evolutions of the creep compliances for loading at concrete ages of 2 days, 7 days, and 28 days are well predicted together with the respective mass water content evolution. This also holds for the evolution of the drying shrinkage strain, at least for moderate drying up to one year. However, it will be demonstrated that for longer drying times further conceptual thoughts concerning the coupled representation of shrinkage and creep are required.

## 1. Introduction

Shrinkage and creep are important phenomena affecting the load bearing capacity, durability and serviceability of plain and reinforced concrete structures. Drying of concrete structures commonly results in restraint shrinkage and will therefore cause compressive as well as tensile stresses in a structure. The tensile stresses, often associated with near-surface regions, can cause material degradation and are for this reason potentially harmful to the integrity of the concrete cover. The material degradation can result in an increased susceptibility to chloride ingress and carbonation, and may therefore cause a significant reduction in durability. Creep may induce significant long-term deformations in concrete structures resulting in prestress losses of prestressed concrete members. This may affect the safety as well as the serviceability of these structures. If concrete structures are loaded at early age, either mechanically or hygrally (by drying), the time-dependency of the material behavior, governed by cement hydration, is also of great importance. The chemical reaction rate in the hydration process depends not only on time but on the hygral and thermal state as well, which can be quite inhomogeneous in concrete structures.

Proper consideration of such coupled processes in numerical simulations requires the application of a coupled hygro-thermo-chemo-mechanical multiphase model [1,2]. An example of the successful application of such an approach is documented in [3], in which a multiphase model is used for predicting the interactions between substrate and hardening overlay in repair problems. Another example is the multiphase simulation of the impact of drying shrinkage on the behavior of concrete structures strengthened by concrete overlays, presented in [4]. A further, very recent work on multiphase simulations of early-age concrete behavior can be found in [5].

The use of such a comprehensive multiphase model is both a blessing and a curse. The capability of considering all the different phenomena comes at the price of a large number of balance and constitutive equations with many parameters to be calibrated. The required experiments include calorimetry tests, tests on mechanical and hygral parameters as well as sorption, shrinkage, and creep tests. Since many of these properties are age-dependent, experiments have to be performed not only once but several times at different ages and/or for long durations. Furthermore, they have to be carried out in different ambient conditions.

A major contribution of this paper is the simultaneous calibration of all parameters of a fully coupled hygro-thermo-chemo-mechanical multiphase model for a single concrete mixture, summarized in Table 1, as it is used for concrete overlays. The long-term goal is to apply the developed and calibrated model to tasks in engineering practice, including the simulation of the rehabilitation of a bridge deck, investigated experimentally in [6]. The concrete mixture was therefore selected specifically to resemble the overlay concrete used in that application. This includes the use of a blended cement CEM II/A-M, characterized by a high Portland cement clinker content, and slag and limestone as additional components. Plasticizer and air-entraining agents are also included in the mixture to meet the requirements for an overlay concrete concerning frost resistance and flowability. For the respective concrete mixture a comprehensive set of experimental data exists [7,8,9,10], which will be exploited for calibration. The concrete grade is C30/37 [8]. The grade was determined in accordance with ONR 23303 [11] using three cubic specimens.

The number of different experiments is considerably more comprehensive than the tests used, for instance, for calibrating the material parameters of a multiphase model for shotcrete [12]. Moreover, in contrast to [3], desorption isotherms will be calibrated for the particular concrete. In the present contribution data from calorimetry tests, drying shrinkage tests on thin concrete slices, and accompanying water content measurements in sealed and unsealed samples will be exploited, i.e., extra information which has not yet been exploited in comparable multiphase simulations like the one in [5]. In addition to data from basic creep tests, data from drying creep tests will be employed for calibration.

A further key aspect of the present paper is the proposition of a porosity-dependent desorption isotherm based on an analogy to a deformation-dependent soil water retention curve developed in the context of geomechanics [13]. Due to the dependence of porosity on the degree of hydration, the resulting approach is closely related to the one proposed by Sciumé et al. [3], who assumed a desorption isotherm depending on the degree of hydration. Sciumé et al. calibrated their newly introduced parameters from autogenous shrinkage tests. They justified their approach by a comparison of desorption isotherms of ordinary and high-performance concrete, which are associated with different pore structures. However, Sciumé et al. [3] did not have access to experimental desorption isotherms for their particular repair concretes. In contrast, in the present contribution all necessary information is available from test data, including desorption tests as well as an autogenous shrinkage test with accompanying water content measurements. Thus, the porosity-dependent desorption isotherm is fully calibrated from the experimental data taking into account the evolution of porosity.

The paper is structured as follows. Section 2 provides an overview of the governing balance and constitutive equations and the respective parameters to be calibrated in the following sections. The extensive calibration process is described in Section 3. It will be demonstrated that the consistent set of material parameters, obtained by the thorough calibration of all parameters of a multiphase concrete model from the comprehensive test data for the particular concrete, will greatly enhance the predictive capabilities of the coupled model. Finally, a short summary of the calibration procedure is provided in Section 4, and conclusions are drawn in Section 5.

## 2. Basic Equations of the Multiphase Concrete Model

### 2.1. Balance Equations

The hygro-thermo-chemo-mechanical model is governed by a set of balance equations for momentum, mass, and enthalpy, see [1,14,15], for instance. For the following investigations, it is a reasonable simplification to neglect effects of gravity. Furthermore, a passive gas phase is assumed, i.e., the gas pressure $p^{g}$, which is the sum of dry air pressure $p^{\mathrm{ga}}$ and vapor pressure $p^{\mathrm{gw}}$, is constant and equal to the atmospheric pressure $p^{\mathrm{atm}}$. The resulting simplified equations are summarized briefly in this section.

The balance of momentum for the multiphase continuum can be stated for quasi-static conditions using the total stress σ as

(1) ∇∘σ=0.

The balance of mass of the solid phase is given as

(2) $$\frac{d}{dt}\left(\left(1-n\right)\;\cdot \;\rho_{s}\right)+\left(1-n\right)\;\cdot \;\rho_{s}\;\cdot \;\left(\nabla \circ \dot{u}\right)=\dot{\Gamma }\;\cdot \;\Delta m_{w},$$

with the displacement vector u, the porosity *n*, the density of the solid phase $\rho_{s}$, and the ultimate amount of chemically bound water per unit volume $\Delta m_{w}$; 0≤Γ≤1 denotes the normalized degree of hydration as defined in [16]. The term porosity refers to a volume fraction defined in the context of the theory of porous media, i.e., to the ratio between void volume and total volume of a representative volume element.

Based on the passive air phase assumption, and neglecting gravity, the balance of mass of the water vapor phase reads

(3) $$\begin{array}{c} \frac{d}{dt}\left(n\rho_{\mathrm{gw}}\left(1-S_{w}\right)\right)+n\rho_{\mathrm{gw}}\left(1-S_{w}\right)\;\cdot \;\left(\nabla \circ \dot{u}\right)-\nabla \circ \left(\rho_{g}D_{g}^{\mathrm{gw}}\;\cdot \;\nabla \left(\frac{\rho_{\mathrm{gw}}}{\rho_{g}}\right)\right)={\dot{m}}_{\mathrm{vap}}. \end{array}$$

In (3) $\rho_{\mathrm{gw}}$ and $\rho_{g}$ are the densities of water vapor and gas, $S_{w}$ denotes the degree of water saturation, and $D_{g}^{\mathrm{gw}}$ is a diffusion coefficient. As in [4,17], for instance, the latter is assumed to depend on saturation, porosity, and temperature *T*:

(4) $$D_{g}^{\mathrm{gw}}=D_{g,0}^{\mathrm{gw}}\;\cdot \;{\left(\frac{T}{273K}\right)}^{5/3}\;\cdot \;f_{S}\left(n,S_{w}\right),\;f_{S}\left(n,S_{w}\right)=n^{a_{\mathrm{fS}}}\;\cdot \;{\left(1-S_{w}\right)}^{b_{\mathrm{fS}}}.$$

The exponents $a_{\mathrm{fS}}$ and $b_{\mathrm{fS}}$, defining the resistance factor $f_{S}$, can be fitted from the observed transport properties at low saturation [4,18]. The value $D_{g,0}^{\mathrm{gw}}=2.58\times 10^{-5}\;m^{2}/s$ [17] will be used in the following. The mass rate of vaporizing water ${\dot{m}}_{\mathrm{vap}}$ reappears as a sink term in the balance of mass of the liquid water phase

(5) $$\begin{array}{c} \frac{d}{dt}\left(n\rho_{w}S_{w}\right)+n\rho_{w}S_{w}\;\cdot \;\left(\nabla \circ \dot{u}\right)+\nabla \circ \left(\frac{\rho_{w}k_{w}^{\mathrm{rel}}K}{\mu_{w}}\;\cdot \;\nabla p^{c}\right)=-\dot{\Gamma }\;\cdot \;\Delta m_{w}-{\dot{m}}_{\mathrm{vap}}. \end{array}$$

Parameters for the mass flux of liquid water relative to the solid phase are the intrinsic permeability of concrete *K*, the dynamic viscosity of water $\mu_{w}$, and the relative water permeability in a partially saturated state $k_{w}^{\mathrm{rel}}$. The mass flux of liquid water is driven by the gradient of the capillary pressure $p^{c}$. Adding up the mass balance equations of water vapor (3) and liquid water (5) finally yields the total mass balance of the water phase

(6) $$\begin{array}{c} \frac{d}{dt}\left(n\rho_{\mathrm{gw}}\left(1-S_{w}\right)\right)+\frac{d}{dt}\left(n\rho_{w}S_{w}\right)+n\left(\rho_{w}S_{w}+\rho_{\mathrm{gw}}\left(1-S_{w}\right)\right)\;\cdot \;\left(\nabla \circ \dot{u}\right)+\\ +\nabla \circ \left(\frac{\rho_{w}k_{w}^{\mathrm{rel}}K}{\mu_{w}}\;\cdot \;\nabla p^{c}\right)-\nabla \circ \left(\rho_{g}D_{g}^{\mathrm{gw}}\;\cdot \;\nabla \left(\frac{\rho_{\mathrm{gw}}}{\rho_{g}}\right)\right)=-\dot{\Gamma }\;\cdot \;\Delta m_{w}. \end{array}$$

To sum up, the mass balance of the water phase (6) takes into account transport of liquid water according to Darcy’s law and diffusion of water vapor using Fick’s law of diffusion. Hydration extracts water from the mass balance of the water phase (6), which reappears in the balance of mass of the solid phase (2).

The balance of enthalpy for the multiphase mixture is given as

(7) $$\begin{array}{c} C_{p}^{\mathrm{eff}}\rho^{\mathrm{eff}}\;\cdot \;\frac{d}{dt}T+\left(C_{p}^{w}\rho_{w}\left(\frac{k_{w}^{\mathrm{rel}}K}{\mu_{w}}\;\cdot \;\nabla p^{c}\right)\right)\circ \nabla T-\nabla \circ \left(\lambda_{\mathrm{eff}}\nabla T\right)=-\Delta H_{\mathrm{vap}}{\dot{m}}_{\mathrm{vap}}+\dot{\Gamma }\;\cdot \;Q_{\infty }\; \end{array}$$

with

(8) $$C_{p}^{\mathrm{eff}}\rho^{\mathrm{eff}}=\left(1-n\right)\;\cdot \;C_{p}^{s}\rho_{s}+n\;\cdot \;\left[C_{p}^{w}\left(S_{w}\rho_{w}+\left(1-S_{w}\right)\rho_{\mathrm{gw}}\right)+C_{p}^{\mathrm{ga}}\left(\left(1-S_{w}\right)\rho_{\mathrm{ga}}\right),\right]\;$$

denoting the effective heat capacity per unit volume. Here, $C_{p}^{w}$, $C_{p}^{\mathrm{ga}}$, and $C_{p}^{s}$ are the specific heat capacities of water, dry air, and the solid phase, respectively. The value $\lambda_{\mathrm{eff}}$ is the heat conductivity of concrete, $\Delta H_{\mathrm{vap}}$ the enthalpy of evaporation. Furthermore, $Q_{\infty }$ denotes the released heat of hydration per unit volume. The two terms on the right hand side of (7) model cooling by evaporation and heating by cement hydration, respectively. The heat transport by conduction will be dominating the advective transport of heat in many cases. The latter is neglected in related approaches, see [3] for instance. Using the balance of mass of liquid water (5), Equation (7) can be restated in its final form

(9) $$\begin{array}{c} C_{p}^{\mathrm{eff}}\rho^{\mathrm{eff}}\;\cdot \;\frac{d}{dt}T+\left(C_{p}^{w}\rho_{w}\left(\frac{k_{w}^{\mathrm{rel}}K}{\mu_{w}}\;\cdot \;\nabla p^{c}\right)\right)\circ \nabla T-\nabla \circ \left(\lambda_{\mathrm{eff}}\nabla T\right)-\\ -\Delta H_{\mathrm{vap}}\left(\frac{d}{dt}\left(n\rho_{w}S_{w}\right)+n\left(\rho_{w}S_{w}\right)\;\cdot \;\left(\nabla \circ \dot{u}\right)+\nabla \circ \left(\frac{\rho_{w}k_{w}^{\mathrm{rel}}K}{\mu_{w}}\;\cdot \;\nabla p^{c}\right)+\dot{\Gamma }\Delta m_{w}\right)=\dot{\Gamma }\;\cdot \;Q_{\infty }\; \end{array}$$

All time derivatives are evaluated with respect to an observer fixed in the material configuration of the solid phase. The approach used here takes into account full coupling between the hygral and mechanical problem. Nevertheless, the observed back-coupling from mechanical deformation to the hygral problem is weak.

### 2.2. Reaction and Constitutive Models

#### 2.2.1. Hydration Model

The chemical reactions, characterizing the hydration process, are modelled taking into account chemo-hygro-thermal phenomena at a macroscopic level. For that purpose, the reactions are lumped into a single reaction model for a normalized degree of hydration, as also done in [3,5]. The evolution of this normalized degree of hydration is described based on the expression given in [1,19]:

(10) $$\dot{\Gamma }=\frac{A_{1}\;\cdot \;\left(\frac{A_{2}}{\kappa_{\infty }^{w/c}}+\kappa_{\infty }^{w/c}\;\cdot \;\Gamma \right)\;\cdot \;\left(1-\Gamma \right)}{1+625\;\cdot \;{\left(1-\varphi \right)}^{4}}\;\cdot \;\exp\left(-\bar{\eta }\;\cdot \;\Gamma -\frac{E_{a}}{RT}\right).$$

$A_{1}$, $A_{2}$ and $\bar{\eta }$ are the main parameters governing the reaction law. This equation features both, dependence on temperature and dependence on relative humidity φ. The ultimate degree of hydration $\kappa_{\infty }^{w/c}$ is estimated based on the water-cement ratio given in Table 1. For the activation energy divided by the universal gas constant, $E_{a}/R=5000\;K$ is used. This activation energy cannot be interpreted as the activation energy of a single component’s reaction. It is rather a parameter included in the lumped model to acknowledge hydration in general as a thermally activated process.

#### 2.2.2. Evolution of Compressive Strength

Compressive strength evolution was modelled as in [2,20] using a power-law equation:

(11) $$f_{c}\left(\Gamma \right)=f_{c,\;\infty }\;\cdot \;{\left(\frac{\Gamma -\Gamma_{\mathrm{init}}}{1-\Gamma_{\mathrm{init}}}\right)}^{a_{\mathrm{fc}}}.$$

The exponent $a_{\mathrm{fc}}$ is a dimensionless fitting parameter. The hardening concrete is assumed to solidify at the degree of hydration $\Gamma_{\mathrm{init}}=0.1$ [3,21]. The compressive strength reaches its maximum value $f_{c,\;\infty }$ at Γ=1. The long-term evolution of the compressive strength reflects the ongoing hydration process of hardening concrete and can therefore be used for further assessing the validity of the assumed hydration model and its calibration for time periods longer than the ones covered in calorimetry tests.

#### 2.2.3. Desorption Isotherm

In hygral equilibrium and isothermal conditions, the relation between the mass water content $w_{c}$ in a concrete specimen and the ambient relative humidity φ is called a sorption isotherm. It depends on the microstructure of the pore system. If the specimen experienced a decrease in humidity only, one refers to it as a desorption isotherm. The mass water content $w_{c}$ can be related to the degree of water saturation $S_{w}$ by means of the theory of porous media, and the relative humidity φ to the capillary pressure $p^{c}$ via the Kelvin–Laplace law

(12) $$\varphi =\exp\left(-\frac{p^{c}\;\cdot \;M_{w}}{\rho_{w}\;\cdot \;R\;\cdot \;T}\right),$$

with the molar mass of water $M_{w}$. Strictly speaking, Equation (12) holds for pure water contained in the pore space [22]. For a given relative humidity, the presence of dissolved species in the pore fluid may cause a reduction of the capillary pressure in the pore network. As a consequence, the stress which is driving shrinkage deformation in an effective stress shrinkage model would be smaller [22]. Since in the present context no quantitative information is available on the content of dissolved species in the pore fluid, the effect of the presence of dissolved species is not modelled explicitly by a modified version of (12). Rather, the impact of the reduced shrinkage-driving stress on the deformation is taken into account implicitly by the calibration of the parameters $a_{\chi }$ and $b_{\chi }$ of the shrinkage model which will be presented in the next paragraph.

In the present contribution, the desorption isotherm is included in a constitutive equation for the degree of water saturation. Its general form is assumed as:

(13) $$S_{w}=S_{w}\left(p^{c},n\right)\;$$

in terms of the capillary pressure and porosity.

#### 2.2.4. Shrinkage and Creep Models

Shrinkage is modelled following the approach of [1,2]. Assuming a passive gas phase, a generalized effective stress is defined as

(14) $$\sigma^{\mathrm{eff}}=\sigma -1\;\cdot \;\alpha_{\mathrm{Biot}}\;\cdot \;\chi \left(S_{w}\right)\;\cdot \;p^{c}\;.$$

This formulation adopts the generalized saturation-dependent Bishop parameter $\alpha_{\mathrm{Biot}}\;\cdot \;\chi \left(S_{w}\right)$ containing the Biot coefficient $\alpha_{\mathrm{Biot}}$ accounting for the compressibility of the solid phase. A linear dependency of the Bishop parameter on the degree of water saturation is assumed:

(15) $$\alpha_{\mathrm{Biot}}\;\cdot \;\chi \left(S_{w}\right)=a_{\chi }\;\cdot \;S_{w}-b_{\chi }.$$

The constitutive relation describing the mechanical behavior is assumed to be governed by the effective stress rate, i.e.,

(16) $$\dot{\varepsilon }-{\dot{\varepsilon }}^{\mathrm{th}}-{\dot{\varepsilon }}^{\mathrm{cr}}=E_{\mathrm{eff}}^{-1}G{\dot{\sigma }}^{\mathrm{eff}}\;$$

with $\dot{\varepsilon }$, ${\dot{\varepsilon }}^{\mathrm{th}}$, and ${\dot{\varepsilon }}^{\mathrm{cr}}$ denoting the total, thermal, and creep strain rates, respectively. The matrix G is the elastic compliance matrix of Hooke’s law for a unit elastic modulus. It generalizes the 1D creep formulation to 3D and reads

(17) $$G=\left[\begin{array}{cccccc} 1 & -\nu & -\nu & 0 & 0 & 0\\ -\nu & 1 & -\nu & 0 & 0 & 0\\ -\nu & -\nu & 1 & 0 & 0 & 0\\ 0 & 0 & 0 & 2\left(1+\nu \right) & 0 & 0\\ 0 & 0 & 0 & 0 & 2\left(1+\nu \right) & 0\\ 0 & 0 & 0 & 0 & 0 & 2\left(1+\nu \right) \end{array},\right]$$

with a constant Poisson’s ratio taken as ν=0.2. $E_{\mathrm{eff}}^{-1}$ is the effective elastic-viscoelastic compliance of the model. The shrinkage strain rate ${\dot{\varepsilon }}^{\mathrm{sh}}$ can be introduced to restate (16) in a total stress context, viz.

(18) $$\dot{\varepsilon }-{\dot{\varepsilon }}^{\mathrm{th}}-{\dot{\varepsilon }}^{\mathrm{cr}}-{\dot{\varepsilon }}^{\mathrm{sh}}=E_{\mathrm{eff}}^{-1}G\dot{\sigma }.$$

Therefore, from (16) and (18), the shrinkage strain rate ${\dot{\varepsilon }}^{\mathrm{sh}}$ is defined implicitly based on the effective stress assumption:

(19) $${\dot{\varepsilon }}^{\mathrm{sh}}=E_{\mathrm{eff}}^{-1}G\left({\dot{\sigma }}^{\mathrm{eff}}-\dot{\sigma }\right)\;.$$

A decomposition of the creep strain rate into a viscoelastic part ${\dot{\varepsilon }}^{\mathrm{cve}}$ and a viscous flow part ${\dot{\varepsilon }}^{\mathrm{cf}}$ is assumed, i.e.,

(20) $${\dot{\varepsilon }}^{\mathrm{cr}}={\dot{\varepsilon }}^{\mathrm{cve}}+{\dot{\varepsilon }}^{\mathrm{cf}}.$$

The thermal strain rate is proportional to the rate of temperature, i.e., ${\dot{\varepsilon }}^{\mathrm{th}}=\alpha_{T}\dot{T}\;\cdot \;1$. The thermal expansion coefficient used in the computations is $\alpha_{T}=1.1\times 10^{-5}/K$.

The effective elastic-viscoelastic compliance $E_{\mathrm{eff}}^{-1}$ of the model is composed additively of an asymptotic elastic and a viscoelastic component, the latter depending on the time period of observation:

(21) $$E_{\mathrm{eff}}^{-1}=E_{\mathrm{asym}}^{-1}+E_{\mathrm{ve}}^{-1}.$$

In the formulation of [2] adopted herein, the asymptotic elastic compliance $E_{\mathrm{asym}}^{-1}$, related to the asymptotic instantaneous elastic strain $\varepsilon^{a}$, depends on Γ:

(22) $$\frac{1}{E_{\mathrm{asym}}}=\frac{1}{E_{\mathrm{asym},\;\infty }}\;\cdot \;{\left(\frac{\Gamma -\Gamma_{\mathrm{init}}}{1-\Gamma_{\mathrm{init}}}\right)}^{-b_{E}}.$$

The parameters are the asymptotic elastic compliance $E_{\mathrm{asym},\;\infty }^{-1}$ at Γ=1 and the exponent $b_{E}$. $E_{\mathrm{ve}}^{-1}$ in (21) is the rate- and hydration-dependent effective viscoelastic compliance of the microprestress solidification creep model [23,24] transferred to multiphase materials in the formulation of [2]. This viscoelastic part of the compliance can be derived from the definition of the viscoelastic strain rate:

(23) $${\dot{\varepsilon }}^{\mathrm{cve}}=\frac{1}{\Gamma \left(t\right)}\int_{0}^{t}\frac{d\Phi }{dt}\left(t-t^{\prime }\right)G{\dot{\sigma }}^{\mathrm{eff}}\left(t^{\prime }\right)\;dt^{\prime }.$$

The microscopic creep compliance function of the solidified matter is independent of the degree of hydration and is assumed to be of log-power law type [25] with a compliance parameter $q_{2}$:

(24) $$\Phi \left(t-t^{\prime }\right)=q_{2}\ln\left(1+{\left(\frac{t-t^{\prime }}{1\;\mathrm{day}}\right)}^{0.1}\right).$$

For the implementation, the microscopic creep compliance is expanded into a Dirichlet series [26], consisting in the present case of eight units of a Kelvin chain with the smallest retardation time of $10^{-5}$ days. All age-dependent parameters are kept constant within each time step. See [2] for how the effective elastic-viscoelastic compliance is evaluated in a practical implementation. Using (14), (18), and (19), the constitutive relation describing the mechanical behavior (16) can be reformulated as

(25) $$\dot{\sigma }=E_{\mathrm{eff}}\;\cdot \;G^{-1}\;\cdot \;\left(\dot{\varepsilon }-{\dot{\varepsilon }}^{\mathrm{cr}}-{\dot{\varepsilon }}^{\mathrm{th}}-{\dot{\varepsilon }}^{\mathrm{sh}}\right)\;\;\mathrm{with}\;{\dot{\varepsilon }}^{\mathrm{sh}}=-1\;\cdot \;\frac{1}{3K_{\mathrm{eff}}}\;\cdot \;\frac{d\left(\alpha_{\mathrm{Biot}}\;\cdot \;\chi \left(S_{w}\right)\;\cdot \;p^{c}\right)}{dt}.$$

Here, $K_{\mathrm{eff}}=\frac{E_{\mathrm{eff}}}{3\left(1-2\nu \right)}$ is a rate- and hydration-dependent effective elastic-viscoelastic bulk modulus. In this formulation, viscous and viscoelastic creep are assumed to be driven by the effective stress. The viscous flow part of the creep law is stated based on the microprestress theory as

(26) $${\dot{\varepsilon }}^{\mathrm{cf}}=2cSG\sigma^{\mathrm{eff}}\;$$

and the microprestress *S* evolves according to [23]

(27) $$\dot{S}+c_{0}S^{2}=-c_{1}\;\cdot \;\dot{\left(\ln\varphi \right)}\;,$$

with *c*, $c_{0}$ and $c_{1}$ representing viscous creep parameters. At constant relative humidity, the viscous flow part of the creep law is governed only by a single parameter, the ratio $\frac{2c}{c_{0}}$. It is therefore common to introduce this ratio as an alternative parameter $q_{4}=\frac{2c}{c_{0}}$ [27].

## 3. Parameter Identification

### 3.1. Calibration of Parameters Concerning Water Consumption and Porosity Evolution

The total amount of chemically bound water per unit volume $\Delta m_{w}$ in the balance Equations (2) and (6) is estimated based on measurements of both the total mass water content of the fresh and matured concrete. The mass water content $w_{c}^{\mathrm{fresh}}$ of the fresh concrete, experimentally determined using microwave drying, amounts to 7.2% of the fresh concrete mass [9]. The mass water content of the matured concrete $w_{c}^{\infty }=4.6\%$ is taken from water content measurements by a multi-ring-sensor [9] obtained at the end of the autogenous shrinkage test, i.e., after two years of maturing [8]. The concrete fresh density is $\rho^{\mathrm{fresh}}=2320\;kg/m^{3}$, its final dry density $\rho_{\infty }^{\mathrm{dry}}=2244\;kg/m^{3}$. These values result in

(28) $$\Delta m_{w}=w_{c}^{\mathrm{fresh}}\;\cdot \;\rho^{\mathrm{fresh}}-w_{c}^{\infty }\;\cdot \;\rho_{\infty }^{\mathrm{dry}}=63.8\frac{kg}{m^{3}}\;,$$

corresponding to approximately 17.0% of the cement content per cubic meter.

Following [3], the stoichiometrically estimated theoretical amount of chemically bound water is 22.8% of the cement content in the mixture. The ultimate degree of hydration $\kappa_{\infty }$, i.e., the ratio between the actually and theoretically bound water, amounts to 0.746 for the investigated concrete mixture. The ultimate degree of hydration can also be estimated using the formula by Pantazopoulou an Mills [28], see also [1], as

(29) $$\kappa_{\infty }^{w/c}=\frac{1.031w/c}{0.194+w/c}=0.716\;,$$

which is reasonably close to the value obtained above. The latter value corresponds to the normalized degree of hydration Γ=1.

For the evolution of the porosity *n* in the balance equations of Section 2.1 and in the constitutive Equation (13) a linear relation between *n* and Γ is assumed according to [1,3]:

(30) $$n=n_{\infty }+A_{n}\;\cdot \;\left(1-\Gamma \right).$$

The parameters in this equation are $n_{\infty }$, the final porosity, and the parameter $A_{n}$ governing the linear decrease of porosity with increasing hydration. They can be identified using the mass balances of the water and the solid phase, neglecting thermal and mechanical deformation.

In a first step, the porosity $n_{\mathrm{init}}$ at the time when the concrete solidifies, i.e., at $\Gamma_{\mathrm{init}}$, is determined from the mass balance of the water phase (6). It is assumed that prior to becoming a solid all pores are considered to be fully saturated. Thus, the initial mass water content at the time the solid forms is $n_{\mathrm{init}}\;\cdot \;\rho_{w}$. It is equal to the sum of the mass of water remaining in the pores after hydration in a sealed sample, $w_{c}^{\infty }\;\cdot \;\rho_{\infty }^{\mathrm{dry}}$, and the mass of water consumed by the chemical reaction after the mixture became a solid, $\left(1-\Gamma_{\mathrm{init}}\right)\;\cdot \;\Delta m_{w}$. This yields

(31) $$n_{\mathrm{init}}=\frac{1}{\rho_{w}}\;\cdot \;\left(w_{c}^{\infty }\;\cdot \;\rho_{\infty }^{\mathrm{dry}}+\left(1-\Gamma_{\mathrm{init}}\right)\;\cdot \;\Delta m_{w}\right)=16.1\%.$$

Since according to the mass balance Equations (2) and (6) the water consumed by hydration increases the solid mass, the balance of mass for the solid can be stated in the form

(32) $$\left(1-n_{\infty }\right)\rho_{s}=\left(1-n_{\mathrm{init}}\right)\rho_{s}+\left(1-\Gamma_{\mathrm{init}}\right)\;\cdot \;\Delta m_{w}.$$

The final mass of solid on the left side of this equation is equal to the mass of the solid phase at the time the solid forms plus the amount of water bound in the solid in the subsequent chemical reaction. The density of the solid phase $\rho_{s}$ is assumed to remain constant throughout the reaction process. The mass of the solidified matter rather increases by a decrease in porosity. Therefore, the solid phase density is determined by the final dry density and the final porosity as

(33) $$\rho_{s}=\frac{\rho_{\infty }^{\mathrm{dry}}}{1-n_{\infty }}.$$

In combination with (32) one obtains

(34) $$\rho_{s}=\frac{\rho_{\infty }^{\mathrm{dry}}-\left(1-\Gamma_{\mathrm{init}}\right)\;\cdot \;\Delta m_{w}}{1-n_{\mathrm{init}}}=2606.1\;\frac{kg}{m^{3}}$$

and

(35) $$n_{\infty }=1-\frac{\left(1-n_{\mathrm{init}}\right)\;\cdot \;\rho_{\infty }^{\mathrm{dry}}}{\rho_{\infty }^{\mathrm{dry}}-\left(1-\Gamma_{\mathrm{init}}\right)\;\cdot \;\Delta m_{w}}=13.89\%.$$

From the latter and the value for $n_{\mathrm{init}}$ according to (31), the parameter $A_{n}$ in (30) can be determined as

(36) $$A_{n}=\frac{n_{\mathrm{init}}-n_{\infty }}{\left(1-\Gamma_{\mathrm{init}}\right)}=2.45\%.$$

The balance of mass of the solid phase (2) allows a further reinterpretation of $A_{n}$. Neglecting thermal and mechanical deformation, (2) yields together with (30)

(37) $$\frac{d\left(1-n\right)\rho_{s}}{dt}=-\frac{dn}{dt}\rho_{s}=A_{n}\frac{d\Gamma }{dt}\rho_{s}=\frac{d\Gamma }{dt}\;\cdot \;\Delta m_{w}.$$

Therefore, $A_{n}$ can be equally expressed as

(38) $$A_{n}=\frac{\Delta m_{w}}{\rho_{s}}=\Delta m_{w}\;\cdot \;\frac{\left(1-n_{\infty }\right)}{\rho_{\infty }^{\mathrm{dry}}}.$$

For the present model, the parameters $n_{\infty }$ and $A_{n}$ are fully determined by the mass water content of the fresh concrete, by the ultimate mass water content obtained from a sealed sample, and by the ultimate dry density. Nevertheless, the computed value of $n_{\infty }$ in (35) is close to the measured value of 13.4% obtained experimentally 56 days after casting. Furthermore, these values can also be compared to values obtained for $n_{\infty }$ and $A_{n}$ based on stoichiometric considerations for cement paste [29], as proposed in [30]. According to this approach, the final porosity of the cement paste (CP) is obtained as

(39) $$n_{\infty }^{\mathrm{CP}}=p^{\mathrm{CP}}-0.52\;\cdot \;\left(1-p^{\mathrm{CP}}\right)\;\cdot \;\kappa_{\infty }^{w/c}$$

with the initial porosity of the cement paste given as

(40) $$p^{\mathrm{CP}}=\frac{w/c}{w/c+0.3175}.$$

By analogy to (30), the usual linear relation between porosity and normalized degree of hydration is assumed, this time for the cement paste. The corresponding parameter is computed as

(41) $$A_{n}^{\mathrm{CP}}=0.52\;\cdot \;\left(1-p^{\mathrm{CP}}\right)\;\cdot \;\kappa_{\infty }^{w/c}.$$

The parameters for the concrete mixture are then obtained by multiplying $n_{\infty }^{\mathrm{CP}}$ and $A_{n}^{\mathrm{CP}}$ with the volume fraction of the cement paste in the concrete $\Omega^{\mathrm{CP}}$. The latter is estimated as the complement of the volume fraction of the aggregates, which is obtained using the aggregate content given in Table 1 and an assumed density of the aggregates of 2750 $kg/m^{3}$ as $\Omega^{\mathrm{CP}}=33.9\%$. Hence,

(42) $$n_{\infty }^{\mathrm{stoich}}=n_{\infty }^{\mathrm{CP}}\;\cdot \;\Omega^{\mathrm{CP}}=14.4\%,\;A_{n}^{\mathrm{stoich}}=A_{n}^{\mathrm{CP}}\;\cdot \;\Omega^{\mathrm{CP}}=5.3\%.$$

The final value of the porosity is reasonably close to the value obtained in (35). However, the change in porosity with increasing degree of hydration is considerably higher according to the approach in [30]. It appears that the assumption of a constant solid skeleton density, which is used in [1] and in the present paper, is not fully compatible with these stoichiometric considerations. This statement is in agreement with the work of Gasch et al. [5], who present a hydration-dependent solid phase density based on a microstructural model considering the different solid constituents.

### 3.2. Calibration of Parameters Related to Reaction Kinetics

**Figure 1:**
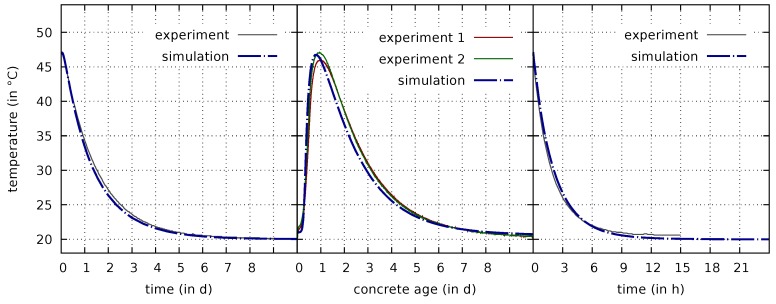
Comparisons of computed and measured temperature evolutions for a matured and preheated insulated cubic specimen (**left**), for a hardening insulated cubic specimen (**center**), and for a matured and preheated cylindrical specimen without insulation (**right**).

Information on the early-age reaction kinetics is obtained from temperature measurements on insulated cubic concrete specimens. For these tests, a cubic specimen with the edge length of 200 mm was enclosed in an extruded polystyrene foam (XPS) formwork with a thickness of 100 mm. A first test is used for calibrating the heat conductivity of the insulation. In this test, a matured specimen, preheated to 47 ${}^{{}^{\circ}}C$, was enclosed in the insulating box, which was then stored in a climatic chamber at 20 ${}^{{}^{\circ}}C$ and the temporal decay of temperature was measured [10]. For the numerical simulation of this test, a constant normalized degree of hydration of Γ=1 is used. The computed temperature evolution using the calibrated heat conductivity for the insulating material of 0.05 W/m K is compared with the measurement data in Figure 1(left).

In a further test, fresh concrete was cast in the insulated box, which again was stored at the constant ambient temperature of 20 ${}^{{}^{\circ}}C$. The temperature evolution, measured in this test [10], can be used for determining the parameters governing the early-age hydration. In this context, the concrete age is referred to the time t=0 when the water was added during the mixing procedure. The hydration process is simulated taking into account only chemo-hygro-thermal phenomena. Therefore, the initial degree of hydration is set to zero (this chemo-hygro-thermal simulation is the only one which is started from Γ=0; all simulations involving the full hygro-thermo-chemo-mechanical coupling are started from the time when the concrete is assumed to become a solid, i.e., at $\Gamma_{\mathrm{init}}=0.1$; this state is reached after 8 hours in the simulation of the second test). The evolution of the normalized degree of hydration is modelled based on the expression (10) together with (29) with the calibrated parameters $A_{1}=1.35\times 10^{4}/s$, $A_{2}=1.0\times 10^{-5}$, $\bar{\eta }=11.0$, and the released heat of hydration of $Q_{\infty }=190\;MJ/m^{3}$, corresponding to 506.7 kJ/kg cement. The respective computed temperature evolution is compared with the measurement data in Figure 1(center). In particular, $\bar{\eta }$ has a strong influence on the long-term response of the hydration model. The value for $\bar{\eta }$ given above is therefore calibrated not only by the calorimetry tests but also by the evolutions of the mechanical parameters (cf. Section 3.3) and the mass water content measured in the autogenous shrinkage test (cf. Section 3.6.2).

From a third test, performed on a matured cylindrical specimen, the convective heat transfer coefficient $\beta_{T}$, required for a convective thermal boundary condition with the convective heat flux $q_{T}=\beta_{T}\Delta T\;\cdot \;n$, is determined. It is required for the following fully coupled hygro-thermo-chemo-mechanical computations. The value $q_{T}$ is proportional to the difference between surface and ambient temperature ΔT and points in the direction of the unit normal n. In this test, the specimen was preheated to 47 ${}^{{}^{\circ}}C$ and then stored in a climatic chamber at 20 ${}^{{}^{\circ}}C$. The measured rapid temperature decay without insulation is shown in Figure 1(right), as well as the simulation results for the calibrated value of $\beta_{T}=9.0\;W/m^{2}\;K$.

### 3.3. Calibration of the Compressive Strength and Elastic Modulus

The parameters for the evolutions of the compressive strength and the elastic modulus are determined from tests on cylindrical and prismatic concrete specimens, respectively. The cylinders used in the compressive strength tests had a diameter of 150 mm and a height of 450 mm. The prims used in the elastic modulus tests were characterized by dimensions of 100 mm × 100 mm × 400 mm. For the computations, the specimens are assumed to be sealed until the beginning of testing at concrete ages of 2 days, 7 days, 28 days, 56 days, 112 days, and 365 days. This corresponds to the test conditions [7,8].

**Figure 2:**
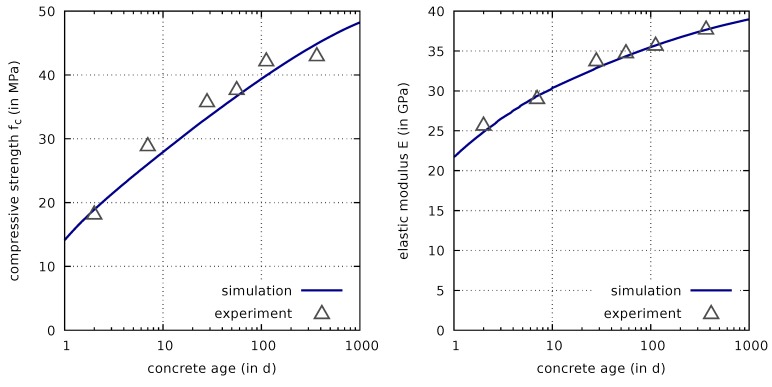
Comparison of computed and measured evolutions of the uniaxial compressive strength (**left**) and the elastic modulus (**right**).

Compressive strength evolution is modelled according to (11). The strength evolution computed on the basis of the identified parameters $a_{\mathrm{fc}}=1.1$ and $f_{c,\;\infty }=51.3$ MPa is compared with the respective measurement data [7,8] in Figure 2(left).

The excellent computed results for the calorimetry test, shown in the previous subsection, indicate that the parameters for the early-age evolution of the degree of hydration are well calibrated. The good agreement with the experimental data visible in Figure 2(left) furthermore confirms that also the long-term evolution of the degree of hydration, which is directly linked to the uniaxial compressive strength evolution according to (11), is well represented by the calibrated model.

The elastic modulus measured in experiments depends on the concrete age as well as on the duration of the measurement period. Therefore, a model for describing the mechanical response of hardening concrete has to take into account such effects. A promising approach to meet these requirements is the use of a multiphase creep model [2] based on the microprestress solidification theory [23,24], described in Section 2.2.4. The elastic modulus tests were performed at constant temperature and for a short time period. Consequently, thermal and shrinkage effects will not affect the computed effective compliance or elastic modulus. The effective compliance is furthermore independent of the viscous creep parameters, which will be calibrated later using data from long-term basic and drying creep tests. The parameters of (22) and (24), fitted from the elastic modulus data, are $E_{\mathrm{asym},\;\infty }=70180$ MPa, $b_{E}=0.16$, and $q_{2}=27\times 10^{-6}/$MPa. Both in the tests and for the calibration, the effective elastic modulus $E_{\mathrm{eff}}$ was determined for a time period of 80 s, corresponding to a loading period of approximately 20 s plus a holding period of 60 s. The results of the calibration, based on the evolution of the degree of hydration, determined from the calorimetry test in the previous subsection, are shown in Figure 2(right). They confirm good agreement with the experimental data.

### 3.4. Modelling and Calibration of a Porosity-Dependent Desorption Isotherm

#### 3.4.1. Desorption Isotherm

Gallipoli et al. [13] suggested a sorption relation for soils depending on the void ratio. In this approach, the air entry value of a van Genuchten type [31] relation is modified by taking into account the dependence on the void ratio. A similar relation is used in the present context, however, assuming that the change in void ratio is not caused by deformation but rather by hydration products decreasing the void volume. This assumption is in line with the approach of Sciumé et al. [3], who suggested a desorption isotherm depending on the degree of hydration. As indicated by Sciumé et al. [3], the hydration- or porosity-dependent formulation can be calibrated using autogenous shrinkage test data. The particular equation proposed here for the general constitutive relation (13) for the degree of water saturation is a modified version of the respective relation for soils from [13]:

(43) $$S_{w}\left(p^{c},n\right)={\left[1+{\left(\frac{p^{c}}{p_{b}^{c}\left(n\right)}\;\cdot \;A_{D}\left(p^{c}\right)\right)}^{n_{\mathrm{Sw}}}\right]}^{-m_{\mathrm{Sw}}},\;A_{D}\left(p^{c}\right)=\exp\left(D\;\cdot \;\frac{p^{c}}{p_{b}^{c}\left(n_{\infty }\right)}\right),$$

with $n_{\mathrm{Sw}}$, $m_{\mathrm{Sw}}$ and *D* denoting dimensionless fitting parameters and $p_{b}^{c}\left(n_{\infty }\right)$ is the air entry value for the matured concrete. The term $A_{D}\left(p^{c}\right)$ can be considered as an option for obtaining a better fit to the experimental data at lower levels of relative humidity. For D=0, the original formulation used by Gallipoli et al. [13] for soils is recovered. The porosity-dependent air entry value is adopted from the respective relation for soils and reads

(44) $$p_{b}^{c}\left(n\right)=p_{b}^{c}\left(n_{\infty }\right)\;\cdot \;{\left[\frac{\left(1-n\right)\;\cdot \;n_{\infty })}{n\;\cdot \;\left(1-n_{\infty }\right)}\right]}^{\psi }.$$

ψ is a further dimensionless fitting parameter. For ψ=0, the original formulation introduced by van Genuchten for soils is recovered. ψ can be estimated on the basis of the autogenous shrinkage test. In a first attempt, it is chosen such that after one year, a relative humidity of roughly 93% is obtained in the sample. The choice ψ=27.0 will be justified by a comparison of computed and measured evolution of the autogenous shrinkage strain in Section 3.6.2.

The application of a modified air entry value for taking into account changes in the microstructure can also be found in other contexts. For instance, in [32] a temperature-dependent air entry value is used for extrapolating a sorption isotherm to the high temperature range. Another example of a porosity-dependent modification of the standard capillary curve is given in [33]. Therein, the air entry value is scaled by a power-law expression based on the ratio between current and final porosity.

#### 3.4.2. Calibration of the Desorption Isotherm Parameters

For the present model, the parameters $n_{\mathrm{Sw}}$, $m_{\mathrm{Sw}}$, *D* and $p_{b}^{c}\left(n_{\infty }\right)$ in (43) are calibrated using the water content measurements on thin concrete slices with dimensions of 110 mm × 110 mm × 20 mm [8], stored at the concrete age of 43 days in desiccators at 43%, 59%, 75%, and 85% relative humidity. Further information is available on the mass water content $w_{c}^{\mathrm{init}}$ at the time when the concrete solidifies. Since all pores are assumed to be fully saturated at that time, it can be determined as

(45) $$w_{c}^{\mathrm{init}}=\frac{n_{\mathrm{init}}\;\cdot \;\rho_{w}}{\rho_{\infty }^{\mathrm{dry}}}.$$

Using (31) this equation results in

(46) $$w_{c}^{\mathrm{init}}=w_{c}^{\infty }+\left(1-\Gamma_{\mathrm{init}}\right)\;\cdot \;\frac{\Delta m_{w}}{\rho_{\infty }^{\mathrm{dry}}}=7.16\%.$$

Furthermore, a (hypothetical) mass water content at Γ=1.0 and full saturation can be obtained from

(47) $$w_{c}^{\infty ,\;\mathrm{sat},\;\mathrm{hyp}}=\frac{n_{\infty }\;\cdot \;\rho_{w}}{\rho_{\infty }^{\mathrm{dry}}}=6.18\%.$$

Since the desorption isotherm is porosity-dependent, the respective degrees of hydration, at which the water contents were measured, must be estimated. For this purpose, simulations of drying of water saturated thin concrete slices [8], stored in the already mentioned desiccators at different values of ambient relative humidity, were carried out. Since for these concrete slices the hydration process was not fully completed at the onset of drying at the concrete age of 43 days, the actual state of hydration needed to be taken into account in the calibration process. The computed degrees of hydration and the corresponding calibrated values for the water content are shown in Table 2 together with the measured values of the water content, taken from [7,8]. The calibrated parameters of (43) are D=0.225, $p_{b}^{c}\left(n_{\infty }\right)=4.6$ MPa, $n_{\mathrm{Sw}}=0.60$, and $m_{\mathrm{Sw}}=0.185$.

**Figure 3:**
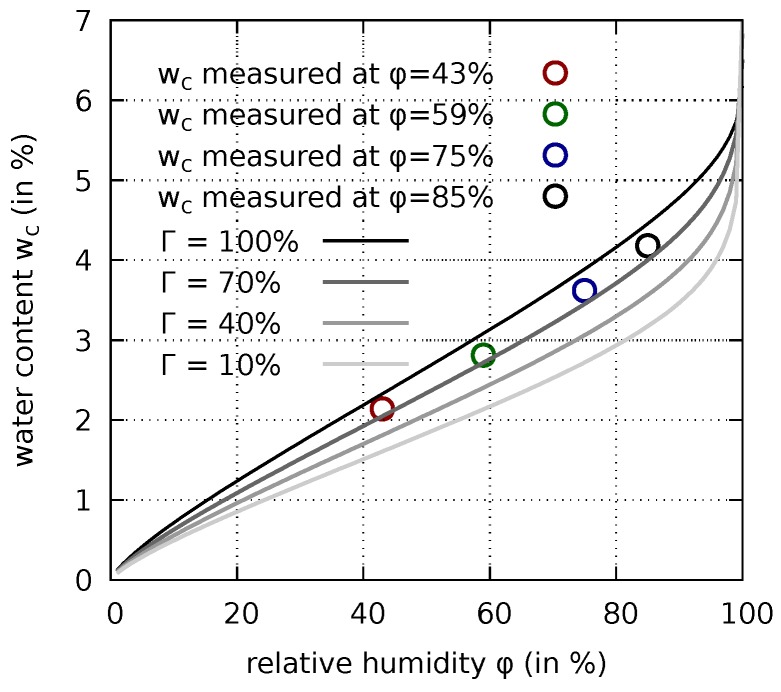
Calibrated desorption isotherm plotted for four different degrees of hydration Γ and four measured points corresponding to three different values of Γ close to 80% (cf. Table 2).

From the calibrated porosity-dependent desorption isotherm representative curves can be obtained for different degrees of hydration as shown in Figure 3 for four selected values of Γ. Note that there is not a single curve corresponding to a unique degree of hydration which passes through all four measured points in this figure. The measured points rather lie on three different curves corresponding to the degrees of hydration of 78%, 80%, and 82%, respectively.

**Figure 4:**
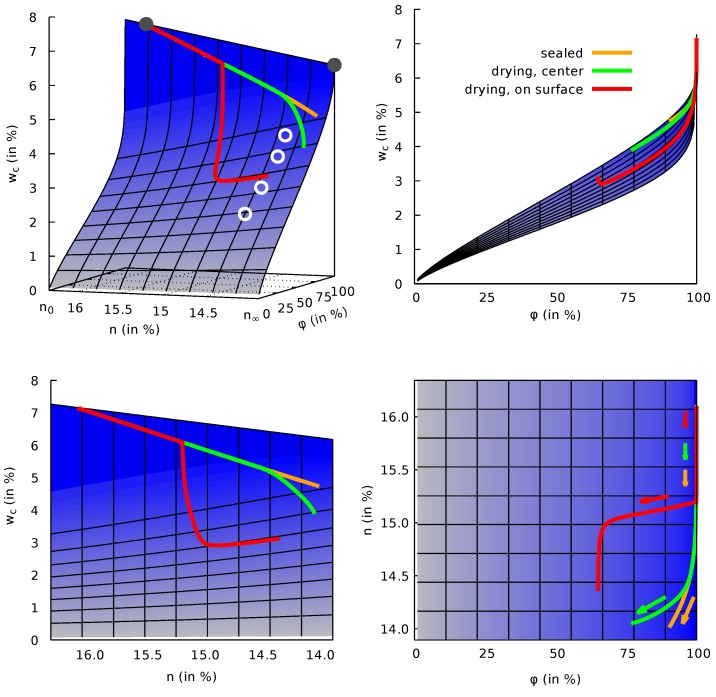
Three-dimensional representation of the desorption isotherm surface in terms of the mass water content $w_{c}$, the porosity *n* and the relative humidity φ (**top left**); side view (**top right**); front view (**bottom left**); top view (**bottom right**). White circles indicate measured values [7,8] whereas grey circles represent estimated values according to (46) and (47).

In Figure 4(top left) a three-dimensional surface plot visualizes the porosity-dependence of the desorption isotherm. This figure contains three paths representing the evolution of the mass water content $w_{c}$, the porosity *n* and the relative humidity φ in a sealed specimen (orange), and at the center (green) and on the surface (red) of a specimen exposed to drying at the age of 2 days. In addition, the four measured points, already shown in Figure 3, are included in Figure 4.

Let us at first focus on the curve, representing the water content evolution resulting from the simulation of a sealed sample. In the first part of this curve the water content mainly decreases by a reduction of porosity while relative humidity remains high. This corresponds to the steep slope of the desorption isotherm close to full saturation for high levels of porosity/low degrees of hydration which can also be seen in Figure 3. Later, the water content change is increasingly governed by a decrease in pore humidity and according to the Kelvin–Laplace Equation (12), capillary pressure will evolve and significant autogenous shrinkage strains will develop.

The water content evolution resulting from the simulation of a sealed sample in Figure 4(bottom left) also reflects the linear dependency between mass water content change and degree of hydration or porosity change for a sealed sample. The other two paths in Figure 4 refer to the simulation of a drying shrinkage test of a cylindrical specimen, which will be addressed in Section 3.6.4.

For all tests the *n*-$w_{c}$-paths start at the location $n_{\mathrm{init}}=16.1\%$ and $w_{c}^{\mathrm{init}}=7.16\%$. The value $w_{c}^{\infty ,\;\mathrm{sat},\;\mathrm{hyp}}=6.18\%$ from (47) is visible in Figure 4(bottom left) as the upper right corner of the desorption surface projected into the *n*-$w_{c}$-plane.

### 3.5. Calibration of Permeability Parameters

According to (6), the transport of liquid water is governed by the product of relative permeability $k_{w}^{\mathrm{rel}}$ and intrinsic permeability *K*. The former corresponds to a reduction factor between zero and one reducing water permeability in partially saturated states, the latter is a degree-of-hydration-dependent material parameter of the porous solid.

The relative permeability is assumed to be governed by a van Genuchten–Mualem based approach [31,34]:

(48) $$k_{w}^{\mathrm{rel}}\left(S_{w}\right)=\sqrt{S_{w}}\;\cdot \;{\left[1-{\left(1-S_{w}^{1/m_{\mathrm{kw}}}\right)}^{m_{\mathrm{kw}}}\right]}^{2}.$$

The dimensionless parameter $m_{\mathrm{kw}}$ can be calibrated in combination with the parameters $a_{\mathrm{fS}}$ and $b_{\mathrm{fS}}$ in (4) based on the mass water content evolution of the thin concrete slices, mentioned in the previous subsection, which were dried at different levels of relative humidity. This yields $m_{\mathrm{kw}}=0.43$, and for the parameters governing the diffusive transport of water vapor $a_{\mathrm{fS}}=2.0$ and $b_{\mathrm{fS}}=4.5$.

In addition, water transport is also studied in cylindrical concrete specimens with 150 mm diameter and 450 mm height for determining the intrinsic permeability. To this end, the mass water content, measured by a multi-ring-sensor embedded at the center of the cylindrical specimens, is exploited [9].

Since transport processes are considered in hardening concrete, for which the structure of the pore system continuously changes due to hydration, the effect of hydration on the intrinsic permeability needs to be considered in the model. Therefore, according to Gawin et al. [1], the intrinsic permeability is approximated as:

(49) $$K\left(\Gamma \right)=K_{\infty }\;\cdot \;10^{A_{\mathrm{perm}}\left(1-\Gamma \right)}.$$

The dimensionless parameter $A_{\mathrm{perm}}$ and the asymptotic intrinsic permeability $K_{\infty }$ are identified as $A_{\mathrm{perm}}=2.5$ and $K_{\infty }=1.45\times 10^{-22}\;m^{2}$ taking additionally into account the shrinkage strain evolution in the drying shrinkage tests, which were started at different concrete ages (cf. Section 3.6.4).

### 3.6. Calibration of the Coupled Shrinkage and Creep Formulation

#### 3.6.1. Identification of the Parameters of the Generalized Effective Stress

**Figure 5:**
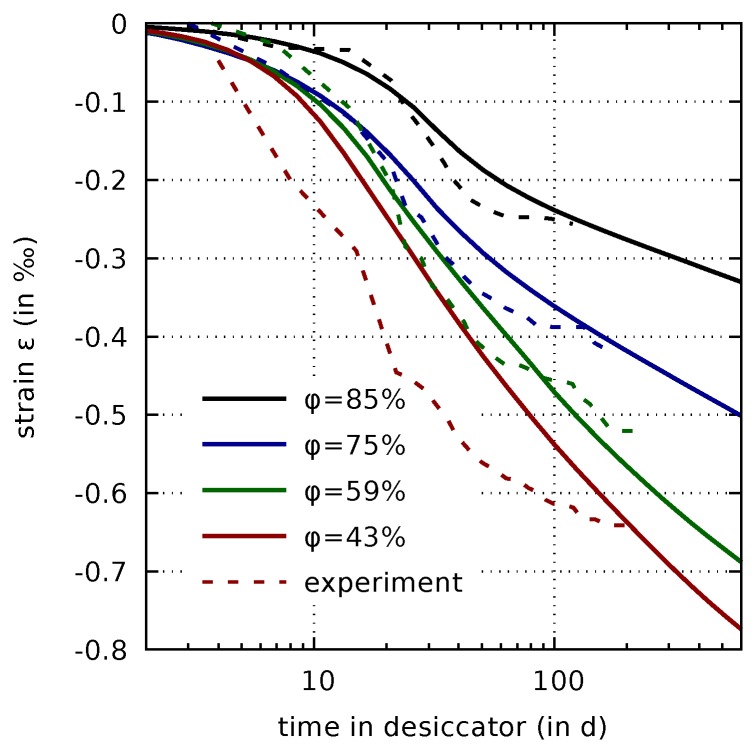
Comparison of drying shrinkage strains measured on thin concrete slices (dashed lines) with the respective computed results.

The constants $a_{\chi }$ and $b_{\chi }$ in (15), which control the internal stress exerted on the porous medium by capillary pressure, are calibrated from the drying shrinkage strain data obtained from drying of the thin concrete slices in the sorption experiments [7], already used in Section 3.4. The identified values for generating Figure 5 are $a_{\chi }=0.67$ and $b_{\chi }=0.08$.

From Figure 5 for the drying levels of φ=85% to φ=59% good agreement between measured and computed evolutions of the drying shrinkage stains can be stated throughout the drying times considered in the experiments. However, for the lowest drying level of φ=43% a further increase of the predicted shrinkage strain after a long drying period can be stated, even after the measured mass water content has attained its final value [7] and, thus, the evolution of the measured shrinkage strain approaches a final value. This discrepancy is the consequence of the creep formulation driven by the effective stress. Remedies of this effect will be proposed in a forthcoming paper.

#### 3.6.2. Calibration of the Parameter for the Early-Age Autogenous Shrinkage

**Figure 6:**
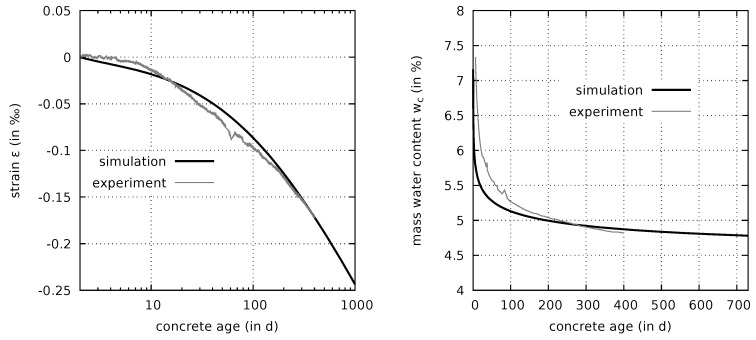
Comparison of measured [7] and computed evolution of the autogenous shrinkage strain (**left**) and of the mass water content in a sealed specimen (**right**).

The fitting parameter ψ in the relation for the porosity-dependent air entry value (44) was initially estimated in Section 3.4.1 as ψ=27.0. It determines the slope of the curve representing the autogenous shrinkage strain for the first few days. It is the only parameter which will be calibrated from measured autogenous shrinkage strains. For this purpose an autogenous shrinkage test [7], performed on a sealed cylindrical specimen with 150 mm in diameter and 450 mm in height, is simulated. The specimen was equipped with a central multi-ring-sensor for indirect measurement of the mass water content [7]. The ambient temperature is 293 K, the heat transfer coefficient, determined in Section 3.2, is taken in the simulation as $\beta_{T}=9.0\;W/m^{2}K$. The comparison of the computed autogenous shrinkage strains with the respective experimental data is shown in Figure 6(left), starting at the concrete age of 2 days.

The predicted values of the relative pore humidity and of the normalized degree of hydration after one year are φ=92.8% and Γ=0.9, respectively. The predicted ongoing hydration process results in a continuous increase of the autogenous shrinkage strain after one year, which is perfectly in line with the trend present in the experimental data.

Figure 6(right) contains a comparison of the computed evolution of the mass water content of a sealed specimen with the respective experimental data. It is emphasized that the continuous decrease in mass water content of the sealed specimen at higher concrete ages is not caused by an insufficient sealing [10]. Rather, the mass water content evolution obtained from the simulation furthermore confirms the well predicted long-term evolution of the degree of hydration. For early-age concrete, Figure 6(right) exhibits a significant discrepancy between the experimentally determined mass water content and the simulation results. This discrepancy does not necessarily indicate an error in the model but can be explained by the way the mass water content is determined in the experiment. It is measured using multi-ring-sensors, i.e., devices which determine the electrolytic resistance of the adjacent material. The resistance is then related to a mass water content based on a calibration curve which is determined for a matured concrete. Therefore, the early-age mass water content measurements can be considered only in a qualitative manner [9].

The corresponding porosity after one year is n=14.1%, see also Figure 4, in which the autogenous shrinkage path is visualized on the porosity-dependent desorption-surface. The good correspondence of measured and computed response confirms the initial estimate ψ=27.0. Thus, an iteration loop with an improved value of ψ can be omitted in the present case.

#### 3.6.3. Calibration of the Parameters of the Viscous Creep Formulation

The parameters for the viscous creep model are calibrated from experiments on cylindrical specimens of the same shape and size as used in the previous paragraph. They were loaded at concrete ages of 2 days, 7 days, and 28 days to 5.49 MPa, 8.7 MPa, and 10.77 MPa, respectively. This corresponds to 30% of the respective uniaxial compressive strength at loading.

The parameters are identified based on measured basic creep and drying creep compliances. The former are obtained from sealed specimens. For the latter, the specimens are only sealed at the beginning and the sealing of the perimetral surface is removed upon load application. The measured compliances are obtained as the difference between measured total strain (obtained from loaded specimens) and measured shrinkage strain (obtained from companion shrinkage tests on load-free samples), normalized by the applied load. The computed compliances are obtained as the difference between computed total strain (obtained from loaded specimens) and computed shrinkage strain (obtained from a companion simulation of a load-free, drying sample), normalized by the applied load. Since calibration is performed by means of the creep compliances only, shrinkage does not affect the calibration of the viscous creep parameters. Therefore, the viscous creep parameters do not depend on the actual calibration of the shrinkage formulation. However, in the creep formulation in terms of the effective stress, this is not true in the reverse direction. The calibration of the viscous creep parameters does affect the shrinkage parameter calibration.

**Figure 7:**
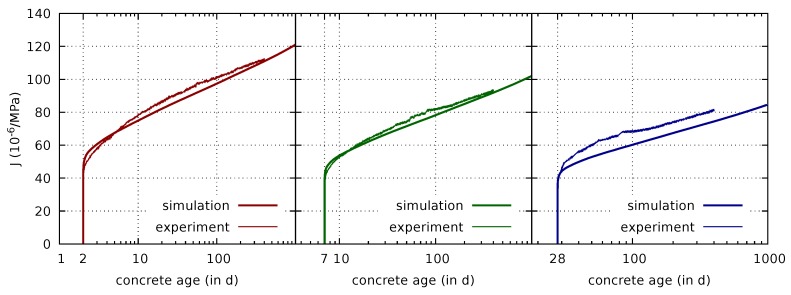
Comparison of measured [7] and computed basic creep compliances for loading at concrete ages of 2 days (**left**), 7 days (**center**), and 28 days (**right**), respectively.

The first value to be set for the creep model is the initial value $S_{0}$ for the microprestress *S* in (26). It is set to 625 MPa at time $t_{0}=1$ day, a suitably chosen time which precedes loading and the onset of drying. Following [27], this initial value determines the model paramater $c_{0}$ in (27). Using the identity $S_{0}=\frac{1}{c_{0}t_{0}}$ results in $c_{0}=1.6\times 10^{-3}/(\mathrm{MPa}\cdot \mathrm{day})$. Following [35,36], the long-term basic creep behavior is governed by the single parameter $q_{4}$ only, which is equivalent to the ratio $\frac{2c}{c_{0}}$ [27]. This parameter is finally calibrated from the measured basic creep compliance curves as $q_{4}=7.9\times 10^{-6}/$MPa. A comparison of the measured basic creep compliance curves with the respective computed curves is displayed in Figure 7. It confirms that the basic creep compliance is well predicted by the multiphase model for different concrete ages at loading.

**Figure 8:**
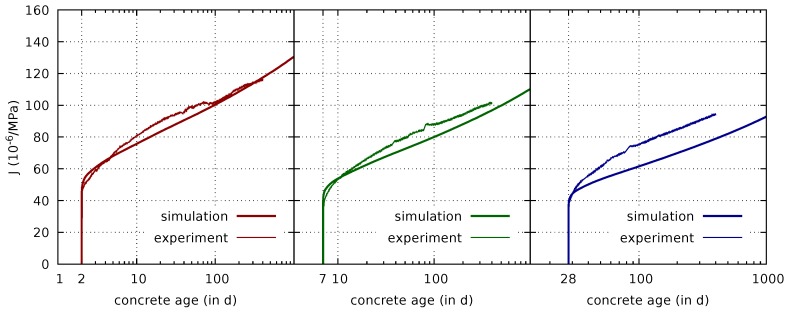
Comparison of measured [7] and computed drying creep compliances for loading and the beginning of drying at concrete ages of 2 days (**left**), 7 days (**center**), and 28 days (**right**), respectively.

The drying creep tests were used to calibrate a single missing parameter of the creep model, the parameter $c_{1}$ in (27), which governs the delay in the decay of the microprestress upon drying. It is fitted using the measured drying creep compliances. The results in Figure 8 are obtained for $c_{1}=10$ MPa.

Since even for sealed samples the internal relative humidity changes due to hydration, the parameter $c_{1}$ has a minor effect on the calibration of the basic creep parameters. The results shown in Figure 7 are obtained for the above given value of $c_{1}=10$ MPa.

#### 3.6.4. Confirmation of the Calibrated Permeability and Shrinkage Parameters by Simulations of Drying Tests on Concrete Cylinders

For confirming the calibrated permeability and shrinkage parameters drying tests on cylindrical concrete specimens [7] with 150 mm diameter and 450 mm height are simulated. The specimens were exposed to drying at concrete ages of 2 days, 7 days, and 28 days, respectively. Drying is modelled following the approach of Sciumé et al. [3] by assuming the water mass flux $q_{W}=\beta_{W}\Delta p^{c}\;\cdot \;n$ across the drying surface as proportional to the difference $\Delta p^{c}$ of the capillary pressure on the surface and the capillary pressure resulting from the ambient humidity of 65% at the ambient temperature of 293 K.

**Figure 9:**
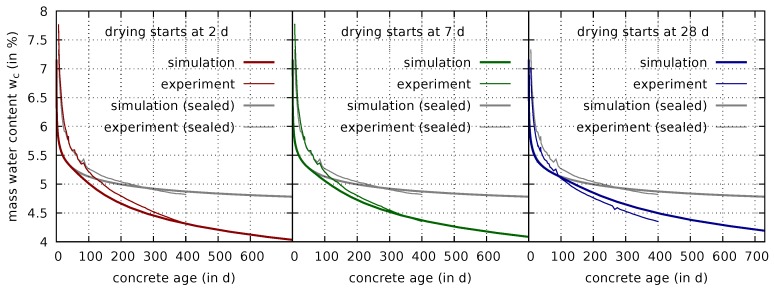
Comparison of measured [7] and computed evolutions of the mass water content in sealed (cf. Figure 6(right)) and in drying cylindrical specimens with drying started at concrete ages of 2 days (**left**), 7 days (**center**), and 28 days (**right**).

The computed evolution of the mass water content for the drying specimens, using the proportionality-coefficient $\beta_{W}=5\times 10^{-14}\;kg/s\;m^{2}$, is shown in Figure 9.

**Figure 10:**
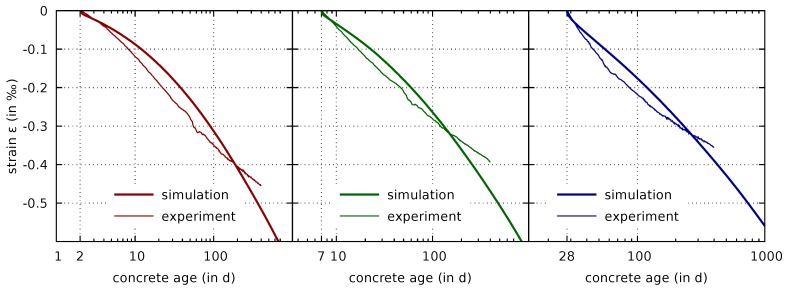
Comparison of measured [7] and computed evolutions of shrinkage strains of drying cylindrical specimens with drying started at concrete ages of 2 days (**left**), 7 days (**center**), and 28 days (**right**).

It follows from Figure 9 that the time when the curves of the drying and sealed specimens deviate from each other is reproduced correctly by the numerical model. Furthermore, the final mass water content, measured for the drying specimens after one year, is also well reproduced by the multiphase simulation. The predicted shrinkage strains are shown in Figure 10.

Recall that the respective experimental results were used for calibrating the permeability parameters in Section 3.5. However, they were not used for calibrating the generalized effective stress parameters, which are the main parameters of the shrinkage formulation. The latter were calibrated by the results of drying tests of thin concrete slices. Thus, the good correspondence of measured and computed evolutions of the shrinkage strains of the drying cylindrical specimens confirms the quality of the proposed parameter identification procedure.

The lower right part of each subfigure in Figure 10 confirm the response which was already visible in the computations of drying shrinkage of concrete slices in Figure 5. Although the shrinkage strains are predicted well for one year of drying, the predicted long-term shrinkage strains seem to be increasingly overestimated. Since for ages above 200 days the mass water contents in the specimens are well recovered by the computational model, this error seems to be caused by the shrinkage formulation. As already mentioned, remedies of this shortcoming will be proposed in a forthcoming paper.

The drying process is visualized in the surface plot of the desorption isotherm in Figure 4. One path corresponds to a point on the surface exposed to drying, the other path to a point at the center of the respective specimen. Both are taken from the simulation for which drying starts at the concrete age of 2 days. From the path referring to the point on the surface it can be seen that drying significantly delays the decrease of porosity. This observation is in line with Baroghel–Bouny and Godin [37].

## 4. Summary of the Calibration Steps

The steps of the calibration procedure for the multiphase concrete model are summarized as follows:

1. Estimate according to (28) the finally chemically bound water per unit volume from the fresh and final mass water content, the latter measured in an autogenous shrinkage test. Determine the parameters of the linear relation between porosity and degree of hydration in (30).
2. Calibrate the parameters of the reaction law (10) for the evolution of the degree of hydration by performing a chemo-hygro-thermal simulation of an insulated hardening specimen. Note that only the short-term behavior is directly visible in this test. The long-term reactive behavior is rather visible in the long-term evolutions of mechanical parameters and mass water content in sealed specimens.
3. Calibrate the parameters of the relation (11) for the evolution of the uniaxial compressive strength and the visco-elastic short-term creep parameters in (22) and (24) for the evolution of the elastic modulus. The accurate prediction of the mechanical parameters and of the mass water content in sealed maturing specimens indicates correct fitting of the parameters used in the reaction law (10).
4. Calibrate the parameters for the desorption isotherm in (43) on the basis of (i) measurement data for the mass water content obtained from thin concrete slices exposed to drying and (ii) measurements of the autogenous shrinkage strain. The former is used for determining the parameters such that the measured values for the mass water content are reproduced for the values of porosity determined from the concrete slices dried at different levels of relative humidity. The porosity-dependence of the desorption isotherm is calibrated by the measured evolution of the autogenous shrinkage strain. The latter step requires a reasonable guess for the Bishop parameter and may have to be repeated in the course of the calibration of the shrinkage model.
5. Calibrate the permeability parameters, i.e., the parameters governing the diffusion of water vapor as well as the parameters governing the transport of liquid water. This is done based on (i) the measured mass water content evolution for thin concrete slices dried at different ambient relative humidities, (ii) the mass water content evolution measured at the center of cylindrical concrete specimens exposed to drying. Calibrate the hydration-dependency of the intrinsic permeability (49) by the measured evolution of the shrinkage strain of maturing cylindrical specimens exposed to drying at different concrete ages.
6. Calibrate the parameters for the Bishop parameter (15) on the basis of the shrinkage strain measurements on thin concrete slices.
7. Perform a simulation of the autogenous shrinkage test using the previously calibrated Bishop and desorption law parameters. If necessary, recalibrate the parameter defining the dependence of the desorption isotherm on the porosity in (44) and recalibrate the desorption law (43).
8. Evaluate the compliances of sealed and unsealed specimens loaded in compression at different concrete ages and use the results for calibrating the viscous creep parameters in the microprestress formulation (26) and (27). Since long-term creep is assumed to be driven by the effective stress, every change in the basic creep and drying creep parameters will affect drying shrinkage, i.e., the calibration of the Bishop-parameter.

## 5. Conclusions

In this paper, a calibration procedure for the numerous parameters of a multiphase model describing the coupled hygro-thermo-chemical-mechanical behavior of concrete at early-age is presented. The major contributions of this work are:

1. The complete calibration of a multiphase concrete model is based on a comprehensive data set from a particular concrete mixture. It includes results from calorimetry tests, results from tests for age-dependent mechanical properties, mass water content and corresponding shrinkage measurements, and results from compressive creep tests on sealed and unsealed specimens loaded at different concrete ages. This thorough calibration allows to fully exploit the capabilities of the coupled multiphase approach.
2. A new desorption isotherm is presented with a porosity-dependent (or, equivalently, a hydration-dependent) air entry value and an extra parameter for the improved approximation of the mass water content at low levels of relative humidity and a suitable calibration procedure is deduced. Compared to existing approaches, the proposed desorption isotherm enables considerably improved predictions of early-age autogenous shrinkage strain.
3. Visualizations of φ-*n*-$w_{c}$-paths for the center and the surface of drying cylindrical specimens are provided, which contribute to a better understanding of the connection between the shape of the desorption isotherm and the predicted evolution of the shrinkage strain.
4. A connection between the evolutions of the porosity and of the mass water content in a sealed hardening concrete sample is established. Thereby, a relation between porosity and degree of hydration is established, based on measured mass water contents and using the mass balances of the water and solid phase, neglecting thermal and mechanical deformation. It is consistent with the applied multiphase model.
5. The viscous, long-term creep parameters of the multiphase model are calibrated by creep compliance data only. The added value of this procedure is especially prominent for a creep formulation in terms of the effective stress, for which shrinkage is coupled to creep while the modelled creep compliance is independent of the predicted shrinkage behavior. The short-term elastic-viscoelastic creep parameters are fitted independently using data from tests for the elastic modulus at different concrete ages.
6. A further highlight of the paper is the use of separate experiments for calibrating and confirming the parameters of the shrinkage formulation in terms of the effective stress. The former is based on experiments on thin concrete slices, whereas the latter is based on tests on cylindrical concrete specimens. The added value of confirming identified parameters is further enhanced by simultaneously assessing the quality of the predictions for both the strain and the accompanying mass water content.

The comparisons of measured and computed evolutions of shrinkage strains reveal limitations of the model. It appears that the model in the current form does not work well for predicting the drying shrinkage strain at the lowest level of investigated ambient relative humidity of 43%. However, the model works well for the investigated higher levels of relative humidity, which correspond to the range usually encountered in concrete structures in central Europe. Although a decent approximation of the drying shrinkage strain is obtained for concrete ages up to one year, the drying shrinkage strain is overestimated for longer drying periods. This observation applies to the simulations of both drying concrete slices and drying concrete cylinders. This problem will be further investigated in a forthcoming paper. Therein, an alternative to the shrinkage and creep formulation in terms of the effective stress will be proposed as a remedy for the mentioned problems.

## Figures and Tables

**Table 1:** Composition of the concrete mixture (concrete grade C30/37).

Component	Amount
Cement CEM II A-M (S-L) 42.5 N, *Lafarge*	375 $kg/m^{3}$
Added water (water/cement ratio of 0.44)	165 $kg/m^{3}$
Limestone sand 0/4 mm	810 $kg/m^{3}$
Limestone aggregates 4/8 mm	183 $kg/m^{3}$
Limestone aggregates 8/16 mm	457 $kg/m^{3}$
Limestone aggregates 16/32 mm	367 $kg/m^{3}$
Plasticizer *Proplast 200*	0.6% of cement mass
Air-entraining agent *Proair NVX*	0.045% of cement mass

**Table 2:** Measured versus calibrated mass water content $w_{c}$ of concrete slices at different relative humidities φ.

φ	Measured $w_{c}$	Computed Γ	Calibrated $w_{c}$
43%	2.14%	78%	2.12%
59%	2.81%	80%	2.83%
75%	3.62%	82%	3.62%
85%	4.18%	82%	4.18%
